# Management strategy and novel ophthalmological findings in neonatal severe hypertriglyceridemia: a case report and literature review

**DOI:** 10.1186/s12944-021-01464-2

**Published:** 2021-04-20

**Authors:** Nehal M. El-koofy, Yasmeen A. Abdo, Dina El-Fayoumi, Amanne F. Esmael, Mohamed. A. Elmonem, Zahraa Ezzeldin

**Affiliations:** 1 Department of Pediatrics, Faculty of Medicine, Cairo University, Cairo, Egypt; 2 Department of Ophthalmology, Faculty of Medicine, Cairo University, Cairo, Egypt; 3 Department of Clinical and Chemical Pathology, Faculty of Medicine, Center of Social and Preventive Medicine, Cairo University, 2 Ali Pasha Ibrahim Street, Room 409, Monira, Cairo, 11628 Egypt

**Keywords:** Neonatal severe hypertriglyceridemia, Chylomicronemia, Lipoprotein lipase deficiency, *LPL* gene, Breastfeeding, Optical coherence tomography, Case report

## Abstract

**Background:**

Neonatal severe hypertriglyceridemia is rarely reported in the literature and there is no consensus for hypertriglyceridemia management at this age group.

**Methods:**

The index case is a 4-week-old male infant with severe hypertriglyceridemia accidentally discovered during a circumcision surgery. His clinical and genetic characteristics and his successful management strategy are described. Furthermore, a detailed ophthalmological examination of the proband was conducted at 3 and 6 months of age using Fourier-domain-optical coherence tomography.

**Results:**

Triglycerides level at presentation was extremely high 33,727 mg/dL (380.8 mmol/L). Two sessions of exchange blood transfusion on two consecutive days successfully reduced triglycerides to 382 mg/dL (4.3 mmol/L) with no adverse effects. The infant was discharged 3 days later. At discharge, the mother was advised to continue breastfeeding together with a medium-chain triglycerides formula. Satisfactory growth parameters and lipid profile values were obtained for a follow-up duration of 5 months with no reported attacks of acute pancreatitis. Lipoprotein lipase deficiency was confirmed by the detection of the *LPL* homozygous pathogenic variant c.805G > A; p.(Glu269Lys). Early corneal and macular lesions were detected and persisted on follow-up despite relatively good lipemic control.

**Conclusion:**

This case highlights the importance of the early discovery of severe hypertriglyceridemia during the neonatal period, which is needed for prompt management and prevention of severe complications. Rationalized breastfeeding can be tolerated within the diet plan of the disease with satisfactory outcomes. To our knowledge, it is the first study reporting early corneal and macular affection by severe hypertriglyceridemia in a neonate. Prolonged follow-up is needed to determine the extent of ophthalmological lesions.

## Introduction

Severe hypertriglyceridemia is an extremely rare clinical presentation in the neonatal period [1]. It is characterized by a fasting serum triglycerides concentration over 1000 mg/dL (11.3 mmol/L) [2, 3]. Severe forms of hypertriglyceridemia pose a significant risk of serious acute pancreatitis in neonates, especially when serum triglycerides levels exceed 2000 mg/dL (22.6 mmol/L) [3]. Familial chylomicronemia, mainly caused by lipoprotein lipase deficiency due to biallelic pathogenic variants in the *LPL* gene is the most common genetic cause of severe hypertriglyceridemia. Although, the reported estimated prevalence of familial chylomicronemia is approximately one per million [3]; this is probably an underestimation particularly in populations with high rates of consanguinity. Hypertriglyceridemia is usually silent and commonly pass undiagnosed in the neonatal period to be detected in older children, adolescents, and sometimes during adulthood depending on the severity of the phenotype [4]. Plasmapheresis has been reported as an effective measure in reducing triglycerides levels in adult patients with hypertriglyceridemia [5]. However, given its risk as an extracorporeal technique, exchange blood transfusion is proposed to be a better alternative for depleting triglycerides in this age group [6–8]. Long-term therapy of severe hypertriglyceridemia of genetic background is usually a lifelong persistent effort. Thus, the recent conception of gene-targeted therapies for such conditions may finally provide the promise of a definitive cure [9].

In the current study, a four-week-old newborn with off-the-charts triglycerides levels due to lipoprotein lipase deficiency is described. Salient clinical features, laboratory and genetics data, and novel findings from a detailed ophthalmological examination with Fourier-domain optical coherence tomography are presented. Procedure details for the successful exchange transfusion for managing hypertriglyceridemia in such a young neonate and his outcomes after 5 months of follow-up are also described.

## Methods

The proband is a male newborn, the second sibling of consanguineous marriage, and was born at term by cesarean section after an uneventful pregnancy. He presented to Cairo University Children’s Hospital at 28 days of age with accidentally discovered milky blood during a routine circumcision surgery. The study was approved by the institutional ethics committee and written informed consent was obtained from the legal guardian of the newborn for conducting the genetic confirmation and for publishing.

Genetic confirmation of the infant was performed at 4 m of age. His DNA was assayed for a targeted gene panel for hyperlipidemia using a next-generation sequencing platform (MiSeq, Illumina, San Diego, CA, USA) including the following genes: *ABCA1, ABCG5, ABCG8, ALMS1, APOA1, APOA5, APOC2, APOC3, APOB, APOE, CREB3L3, GCKR, GPD1, GPIHBP1, LDLR, LDLRAP1, LIPA, LIPI, LMF1, LPL, PCDH15, PCSK9, PNPLA2, SLC25A4, TRIB1.* Suspected pathogenic variants were confirmed by Sanger sequencing (ABI 3100, Applied Biosystems, Waltham, MA, USA) in both the proband and his parents.

Since ophthalmological findings in neonatal hypertriglyceridemia apart from lipemia retinalis are not well reported in the literature, a detailed ophthalmological examination by an expert pediatric ophthalmologist at 3 m of age (2 m after diagnosis) and again 3 m later was performed. Anterior segment examination was conducted with a handheld slit-lamp, while cycloplegic refraction was performed after the installation of cyclopentolate 1% eye drops three times in each eye, 5 min apart, then manual refraction was carried out half an hour after the last drops. Dilated fundus examination was performed using a 20 Diopter lens and indirect ophthalmoscope. Fundus photography was done using RetCam® (Clarity Medical Systems Inc., Pleasanton, CA, USA). Further examination of the anterior segment and the retina was performed using Fourier-domain handheld-optical coherence tomography (HH-OCT); (RTVue RT-100®, Optovue Inc., Fremont, CA, USA). The infant was examined while spontaneously sleeping, using topical anesthesia and a speculum. Anterior segment imaging was conducted in room light and without dilatation using an add-on lens of the corneal adaptor module (CAM-L mode: 6.0–2.0 mm). Posterior segment imaging of the macula and the optic nerve were done using the same device after pupil dilatation and removal of the add-on lens.

## Results

### Case presentation and management

The proband’s physical examination at birth was normal and his birth weight was 2.5 kg. He was exclusively breastfed since birth and until presentation. His parents are in apparent good health, and so is his older brother. At the age of 4 weeks, the father sought surgical advice for his infant to be circumcised. The surgeon noticed milky blood from the wound, so he referred the infant for further evaluation to Cairo University Children’s Hospital. The rest of his neonatal history is irrelevant. The mother reported a history of a previous abortion, but with no history of a previous sibling death. The proband’s grandmother from the father’s side had a long history of hyperlipidemia associated with type 2 diabetes and hypertension.

**Fig. 1:**
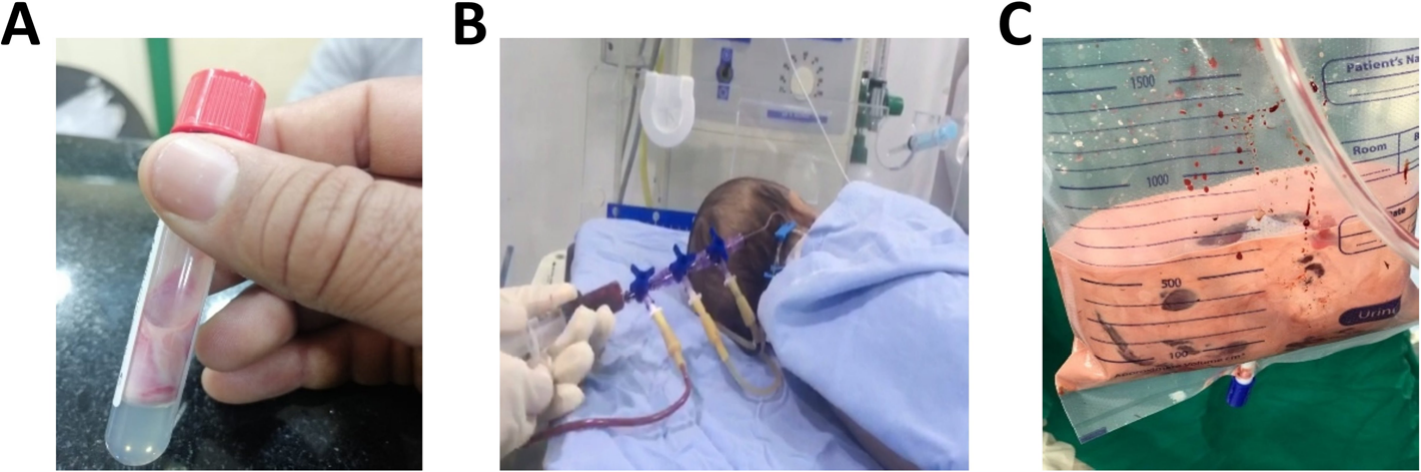
Lipemic blood appearance and exchange transfusion setting. **a** A laboratory test tube showing the pink creamy blood sample on admission. **b** Exchange blood transfusion procedure setting. **c** The milky blood collected in the drainage bag

On admission to the emergency room, the infant was 28 days old. He was vitally stable and active with no clinical evidence of acute pancreatitis. Physical examination showed weight, height, and head circumference within the normal range for age and sex, and no hepatomegaly, xanthomas, xanthelasmas or dysmorphic features. Cardiac and chest examinations were normal. During blood sampling, his blood was highly viscous and pink creamy in color (Fig. 1a). The patient’s lipid profile after proper dilutions revealed extremely elevated serum triglycerides 33,727 mg/dL (380.8 mmol/L), total cholesterol 1844 mg/dL (47.7 mmol/L), high density lipoprotein-cholesterol (HDL) 346 mg/dL (8.9 mmol/L) and low density lipoprotein-cholesterol (LDL) 711 mg/dL (18.4 mmol/L). Lipid profile was assayed twice to confirm the extreme elevation of triglycerides. At presentation liver and kidney functions, complete blood count (CBC) and serum electrolytes couldn’t be evaluated due to the severely lipemic samples. The proband was referred the next day to the neonatal intensive care unit (NICU) for exchange blood transfusion. Written informed consent was obtained from the father for the procedure.

Exchange transfusion was performed twice under complete aseptic conditions to reduce triglycerides to acceptable levels and to minimize the potential for acute pancreatitis. A central venous line was placed in the right internal jugular by surgical cut-down to perform the exchange through the “pull-push technique” using a special 4-way stopcock. The blood bank managed to determine the infant’s blood group (A/Rh + ve), despite his extreme lipemia, and was able to successfully cross match his blood with potential donors. The first exchange was performed on the same day of his referral to the NICU. It lasted approximately three hours using freshly irradiated packed red blood cells suspended in plasma (both RBCs and plasma were A/Rh + ve). Ten ml of the donor’s blood were exchanged with the infant blood each time until a total volume of 700 ml is reached (approximately twofold the estimated infant blood volume with a blood/plasma ratio of 2:1 (Fig. 1b, c). Lipid profile assessment after exchange showed serum levels of triglycerides 5374 mg/dL (60.1 mmol/L), total cholesterol 442 mg/dL (11.4 mmol/L), HDL 135 mg/dL (3.5 mmol/L) and LDL 211 mg/dL (5.5 mmol/L). Since triglyceride values were still unsatisfactory, another blood exchange was arranged the next day. A follow up lipid profile after the second exchange showed much improved serum triglycerides 382 mg/dL (4.3 mmol/L), total cholesterol 198 mg/dL (5.1 mmol/L), HDL 28 mg/dL (0.7 mmol/L) and LDL 94 mg/dL (2.4 mmol/L). Full routine labs were ordered. Serum amylase enzyme activity was completely normal 12 IU/L (Ref. Range 0–96 IU/L), while lipase enzyme activity was slightly elevated 97 IU/L (Ref. Range 0–62 IU/L). CBC, liver and kidney functions, and serum electrolytes were all within normal range. During the patient’s stay in the NICU, a prophylactic course of antibiotics (ampicillin/sulbactam and gentamicin) was given for 5 days until discharge.

The proband was discharged on advice for the mother to continue breastfeeding, constituting approximately 75% of the infant needs in addition to complementary feeding with high medium-chain triglycerides (MCT) formula (monogen®) constituting the remaining 25%. After 2 weeks the ratio was modified to 50%/50% and after 2 months of age, only two night breast milk feedings were allowed (approximately 25%/75% breastfeeding to monogen® ratio). At the age of 4 months, the infant was fully transitioned to monogen®. The fat content of breast milk is quite variable with a range of 1. 5-4.9 g/100 mL. The diet of the mother and parity influence the MCT content of her milk, with a range of 11.4 -13% of the total fat content [10]. In contrast, the monogen® formula contains 2.2 g fat per 100 mL with 84% MCT, thus it should be safer for the infant concerning his lipemic condition. However, the nutritional and immunological values of breast milk are indispensable during this early phase of development, so breast milk was kept in a rationalized form in the infant’s diet during his first 4 months. By 6 months of age, the mother was advised to start weaning with the gradual introduction of puree of vegetables and fruits. Follow up lipid profile was performed at 3 and 6 months of age and revealed satisfactory levels of triglycerides 470 mg/dL (5.3 mmol/L) and 422 mg/dL (4.8 mmol/L), respectively. Table 1 summarizes the lipid profile values performed for the proband during follow-up and for his family during the first visit after discharge. At the age of 6 months, the infant is still having normal growth parameters and didn’t suffer from severe acute pancreatitis.

**Table 1:** Serum lipid profile follow-up of the proband and his family

	Triglycerides mg/dL (mmol/L)	Total Cholesterol mg/dL (mmol/L)	HDL mg/dL (mmol/L)	LDL mg/dL (mmol/L)
**Proband**^a^				
At diagnosis (Day 0)	33,727 (380.8)	1844 (47.7)	346 (8.9)	711 (18.4)
After 1st exchange (Day 1)	5374 (60.7)	442 (11.4)	135 (3.5)	211 (5.5)
After 2nd exchange (Day 2)	382 (4.3)	198 (5.1)	28 (0.72)	94 (2.4)
Day 55	470 (5.3)	160 (4.1)	55 (1.4)	94 (2.4)
Day 150	422 (4.8)	136 (3.5)	28 (0.72)	57 (1.5)
**Family**^b^				
Father	157 (1.8)	185 (4.8)	35 (0.91)	119 (3.1)
Mother	124 (1.4)	171 (4.4)	45 (1.2)	101 (2.6)
Brother	36 (0.41)	127 (3.3)	41 (1.1)	79 (2.0)
Grandmother	785 (8.9)	350 (9.1)	42 (1.1)	151 (3.9)

*HDL* high density lipoprotein cholesterol, *LDL* low density lipoprotein cholesterol, ^a^ The proband was diagnosed on day 28 of his life, ^b^ Family lipid profiles were observed on first visit after discharge of the proband

### Genetic diagnosis

Through NGS a homozygous pathogenic variant was identified in exon-6 of the *LPL* gene (NM_000237.3) c.805G > A; p.(Glu269Lys) and was later confirmed by Sanger sequencing. The variant was segregated in parents according to autosomal recessive inheritance. This variant has been previously described as disease-causing in a Chinese adult in compound heterozygosity with another pathogenic variant p.(Leu279Val) [11], but to the best of our knowledge, it has never been reported in homozygosity or a neonate. This variant was detected as pathogenic by multiple prediction software, such as PolyPhen2, SIFT, MutationTaster, PrimateAI, and varsome. It is also highly conserved among different species and is extremely rare in the gnomAD database with a population frequency of 0.0000119 (3/251,276 alleles).

### Abdominal ultrasonography

At 2.5 months of age (6 weeks after diagnosis) the first abdominal ultrasonography was performed and showed prominent common bile duct with a normal diameter and normal liver size and texture. A second abdominal ultrasonography was performed at 6 months of age and showed a mild but clear echogenic liver.

### Ophthalmological findings

**Fig. 2:**
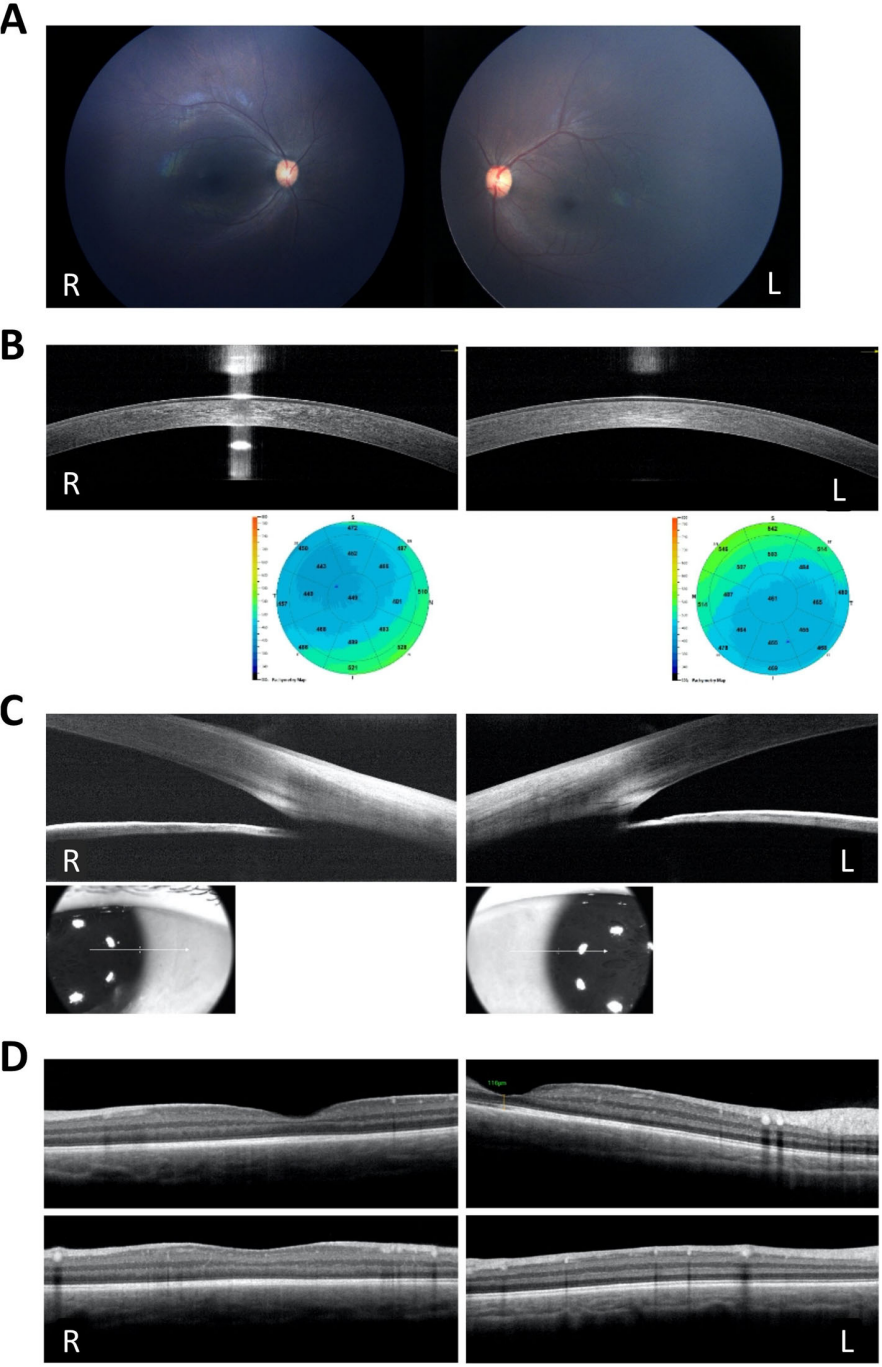
Ophthalmological evaluation of the proband at three months of age. **a** RetCamFundus images showing bilateral normal Right (R) and Left (L) fundi, normal optic nerve head appearance, normal retinal blood vessels and apparently normal macular reflexes. **b** HH-OCT images of the Cornea (Corneal pachymetry map for both eyes using handheld RTVue-ASOCT) showing: Upper: a cross section of the cornea showing subtle hyper-reflective opacities occupying the entire right stroma and subepithelial (R), as compared to the relatively clearer left stroma (L). Lower: Corneal thickness maps showing bilateral central thinning (lower right in both). **c** Imaging of the anterior chamber angle with handheld RTVue-OCT showing normal anterior chamber angle width and structures seen in both eyes. **d** Retina crossline of the macula of both eyes using handheld RTVue-OCT showing hyper-reflective deposits of variable sizes and shapes causing back shadowing and occupying both the superficial and the deep retinal layers. The macula of the left eye shows a wide foveal pit with a central foveal thickness of 116 μm

The first ophthalmological examination was conducted at 3 months of age. It included anterior segment examination, which showed clear corneas and crystalline lens with no evidence of arcus or xanthelasma. Cycloplegic refraction was + 5.00 in both eyes. Dilated fundus examination revealed a dull macular reflex in an otherwise normal fundus (Fig. 2a). Corneal pachymetry (thickness) map (Fig. 2b) showed thinned-out corneas with a central corneal thickness of 448 μm in the right eye and 461 μm in the left eye. Subtle corneal opacities were seen occupying the entire stroma of the right eye (Fig. 2b). Imaging of the anterior chamber angle showed no abnormalities with normal angle structures clearly identified (Fig. 2c). Macular OCT revealed bilateral hyper-reflective deposits of various sizes with backward shadowing (Fig. 2d). These deposits were seen in the deep parts of the retinal nerve fiber layer, ganglion cell layer, inner plexiform layer, inner nuclear layer, and outer plexiform layer. The remaining outer layers of the retina and the choroid were spared. The follow-up ophthalmological evaluation at 6 months of age showed no signs of resolution of both corneal opacities and macular deposits.

## Discussion

The first presentation of neonatal severe hypertriglyceridemia is usually abrupt with recurrent episodes of acute pancreatitis [8, 11–14]. An accidental discovery in the neonatal period, similar to the index proband, is rarely reported, especially with such high triglycerides levels [6]. In the current study, the full clinical data and management strategy of a 4-week-old male who presented with neonatal severe hypertriglyceridemia due to lipoprotein lipase deficiency was described. Significant complications, such as severe acute pancreatitis were successfully prevented during the first 6 months of his life; however, despite the relatively good lipemic control and the absence of lipemia retinalis, ophthalmological abnormalities in the form of persistent corneal and macular deposits were detected by OCT. A mild echogenic liver denoting the start of lipid deposition has also been observed by abdominal ultrasonography.

In the literature, there are several reported management strategies in the early phase of neonatal severe hypertriglyceridemia, but each strategy is only represented by few reported cases. Although invasive, plasmapheresis was tried in neonates following its success in lowering plasma triglycerides efficiently in adult patients and few successful neonatal cases were reported [15, 16]. However, the fear of adverse hemodynamic effects and hemorrhagic events has prevented the routine use of this procedure in the neonatal age group [6]. A more conservative approach using mainly dietary measures, such as complete prevention of breastfeeding, using a dietary formula containing medium-chain triglycerides, and limiting other sources of dietary fats after weaning is followed in many reported cases [12–14, 17, 18]. This approach is usually accompanied by insulin therapy during acute status [12], and sometimes for longer durations after discharge for proper glycemic control and to prevent lipolysis [13, 14, 17]. This approach understandably needs more time to control hypertriglyceridemia and requires fine adjustment of the insulin dose. However, the risk of severe hypoglycemia in a neonate may outweigh the benefits of insulin. More recently, exchange blood transfusion has been proposed as a safer alternative for plasmapheresis for rapidly and effectively depleting extremely high levels of triglycerides in neonates. The procedure although reported in only a few cases [6–8, 19], seemed very efficient with no reported adverse effects. Furthermore, the achieved rapid decline in serum triglycerides allowed us to advise the mother to continue breastfeeding together with the formula, which benefited the infant’s growth and immune system during his early phase of development.

*LPL* gene variants constitute the majority of genetic etiologic factors reported for neonatal cases with severe hypertriglyceridemia; however, many other genes are also involved with this phenotype. They include *GPIHBP1, APOA5, GPD1, APOC2,* and *LMF1*, which produce proteins that are mainly involved in the catabolic pathway of chylomicrons by acting as transporters or chaperones for lipoprotein lipase enzyme [20]. Through the rapid screening of all reported cases of neonatal hypertriglyceridemia with identified genetic etiology published in PubMed over the last 10 years (2011–2020), *LPL* gene variants were responsible for 73% of cases (45/62). Pathogenic variants in *GPIHBP1* gene, which codes for the transporter “glycosylphosphatidylinositol-anchored high-density lipoprotein binding protein-1” were the second most common identified cause and were responsible for almost 16% of cases (10/62) [1, 19, 21]. Deficiency of lipoprotein maturation factor (LMF1) although an established protein in the chylomicrons catabolic pathway was not reported in association with neonatal onset. This may be due to its milder phenotype, which may manifest at a later stage.

Apart from lipemia retinalis, ocular manifestations of severe hypertriglyceridemia are rarely studied, particularly in young infants and children [7, 22, 23]. Ophthalmological examination was not performed at the presentation of the proband before the exchange blood transfusions, thus the presence of lipemia retinalis at this stage cannot be excluded. Furthermore, the retinal lesions detected at 3 and 6 months of age cannot be known for certain whether they are the consequences of the resolution of lipemia retinalis after proper management or did they develop de novo afterward. Capitena et al. reported the rapid and complete resolution of lipemia retinalis in a 7-week old infant after 2 settings of exchange blood transfusion; however, they didn’t report on the development of his ophthalmological condition in the subsequent months [7]. Although serious ophthalmological complications, such as loss of vision were reported in adult familial hypertriglyceridemia patients mainly in association with other known diseases that affect the eye, such as diabetes [24] or hypertension [25], mild ophthalmological sequelae, such as the decreased amplitudes of a-and b-waves in cone and rod responses on electroretinogram have been previously reported [26]. Furthermore, the presence of uncontrolled hypertriglyceridemia is a considerable risk factor for both hyperglycemia and hypertension and thus may pose a significant threat for the eye in the long run. Lipoprotein lipase enzyme activity may also affect the retina differently. Shiba et al. proposed that the enzyme lipoprotein lipase is essential for the normal neurological development of the retina. A reduction of lipoprotein lipase mass and accumulation of visceral fat, as a result, may produce retinal neurodegenerative effects that decrease the retinal nerve fiber layer thickness in adult individuals [27].

To the best of our knowledge, this study is the first to provide a detailed ophthalmological examination of a neonate with severe hypertriglyceridemia and the first to report early persistent corneal and macular lesions detected by OCT despite the proband’s relatively good lipemic control.

## Conclusions

In conclusion, the successful management of a newborn with severe hypertriglyceridemia due to lipoprotein lipase deficiency is presented. The *LPL* pathogenic variant in the proband is reported for the first time in homozygosity associated with neonatal severe hypertriglyceridemia. Two settings of exchange blood transfusion reduced triglycerides to acceptable levels efficiently and rapidly with no adverse effects. Breastfeeding was maintained hand in hand with the medium-chain triglycerides formula with satisfactory outcomes, thus it can be considered in a controlled form within the early phase of the management strategy of neonatal hypertriglyceridemia. Early and subtle ophthalmological lesions have been recorded in the proband. A more prolonged follow-up is needed to determine the long-term ophthalmological effects at this young age.

## Data Availability

All data generated or analysed during this study are included in the published article.

## Abbreviations

HDL: High density lipoprotein-cholesterol
LDL: Low density lipoprotein-cholesterol
LPL: Lipoprotein lipase
NICU: Neonatal intensive care unit
OCT: Optical coherence tomography
